# Reproductive efficacy of a modified-live porcine reproductive and respiratory syndrome vaccine against Korean PRRSV-2 challenge in sows

**DOI:** 10.3389/fvets.2025.1747817

**Published:** 2026-01-05

**Authors:** Jeongmin Suh, Tae-Eun Kim, Sehyeong Ham, Hyejin Na, Ikjae Kang, Sang-Ho Cha, Sun Hee Cho, Chanhee Chae

**Affiliations:** 1 Department of Veterinary Pathology, College of Veterinary Medicine, Seoul National University, Seoul, Republic of Korea; 2 Department of Animal Vaccine Development, BioPOA, Hwaseong-si, Gyeonggi-do, Republic of Korea

**Keywords:** heterologous challenge, modified-live vaccine, porcine reproductive and respiratory syndrome virus, pregnant sow, reproductive failure

## Abstract

**Background:**

Evaluation of the reproductive efficacy of a modified-live vaccine (MLV) based on porcine reproductive and respiratory syndrome virus-2 (PRRSV-2).

**Objective:**

The objective of this study was to evaluate the efficacy of a MLV based on porcine PRRSV-2 against a heterologous Korean PRRSV-2 lineage 1 strain challenge in pregnant sows.

**Animals:**

Twelve pregnant sows from PRRSV-free farms were randomly assigned to three groups (four sows per group).

**Methods:**

Sows in the vaccinated-challenged group received a 2.0 mL intramuscular dose of PRRSV-2 MLV and were subsequently challenged intranasally with a heterologous PRRSV-2 strain.

**Results:**

Vaccinated-challenged sows improved reproductive performance, including a higher number of live-born piglets and fewer stillbirths compared with unvaccinated-challenged sows. Viral genomic loads were lower in vaccinated-challenged sows and their offspring as well. Vaccinated-challenged sows also elicited a significantly higher frequency of PRRSV-2 specific interferon-*γ* secreting cells.

**Discussion:**

These findings demonstrated that the PRRSV-2 MLV administered to pregnant sows provide protection against heterologous challenge through clinical, virological, and immunological evaluation.

## Introduction

Porcine reproductive and respiratory syndrome (PRRS) is one of the most significant infectious diseases in swine, posing a major threat to the global pork industry. It is clinically characterized by respiratory disorders in pigs of all ages and reproductive failure in breeding animals (1). The causative agent, porcine reproductive and respiratory syndrome virus (PRRSV), is an enveloped, single-stranded, positive-sense RNA virus classified within the genus Betaarterivirus, subfamily Variarterivirinae, and family Arteriviridae. PRRSV has been reclassified into two species: Betaarterivirus suid 1 (formerly known as the European genotype or PRRSV-1) and Betaarterivirus suid 2 (formerly the North American genotype or PRRSV-2) (2).

PRRSV-2 was first detected in Korea in 1994 (3) and since its emergence has become the dominant strain circulating within the country’s swine herds (4). Its impact on sows is particularly devastating, as it can cause abortions, stillbirths, and congenital defects in piglets, thereby severely impairing herd reproductive performance (5). Outbreaks of PRRSV-2 in breeding herds have raised serious concerns in the swine industry, primarily due to the enormous economic losses incurred (6, 7). In Korea, where reproductive disorders associated with PRRSV-2 in sows result in particularly severe economic damage, the vaccination of breeding sows is regarded as a critical strategy to mitigate herd-level economic losses and ensure production stability.

Currently, modified-live vaccines (MLVs) are regarded as the most effective tools for controlling the reproductive manifestations of PRRS. These PRRS MLV studies have mainly been limited to piglet evaluation. It is necessary to expand MLV evaluation with breeding sows due to considerable economic damage caused by PRRSV in this population. A commercial PRRSV-2 MLV (registered as PERSOPORC® outside of The Republic of Korea, CEVA Santé Animale, Libourne Cedex, France/POAVAC PRRS® as BIOPOA Co. Ltd., Hwaseong-si, Gyeonggi-do, KOREA within The Republic of Korea) has demonstrated protective efficacy against heterologous PRRSV-2 strains in pigs, particularly in controlling respiratory disease (8). Nevertheless, its experimental efficacy in pregnant sows for the prevention of reproductive disorders has not yet been evaluated. Therefore, the present study was designed to assess the efficacy of PRRSV-2 MLV vaccination in pregnant sows under controlled heterologous challenge conditions.

## Materials and methods

### Ethical statement

All experimental procedures were performed in accordance with the guidelines approved by the Institutional Animal Care and Use Committee of Seoul National University (Protocol No. SNU-240826-3-1).

### PRRSV isolates

PRRSV-2 (SNUVR090851, lineage 1, GenBank no. JN315685) was used as inocula (9).

### Experimental design

Twelve clinically healthy pregnant sows (parity = 2) were obtained from a commercial farm confirmed to be free of PRRSV and with no prior history of PRRSV vaccination. All animals were screeded and tested sera-negative for PRRSV by both enzyme-linked immunosorbent assay (ELISA) and real-time polymerase chain reaction (RT-PCR) (10). The sows were randomly assigned to three treatment groups (*n* = 4 per group), vaccinated and challenged (hereafter called the “Vac/Ch”), unvaccinated and challenged (hereafter called the “UnVac/Ch”), and unvaccinated and unchallenged (hereafter called the “UnVac/UnCh”) groups using the random number generation function in Microsoft Excel (Microsoft Corporation, Redmond, WA, USA). To avoid potential transmission of the vaccine virus between groups, each group was housed in a separate isolation room until the day of viral challenge.

At 21 days prior to challenge (−21 days post challenge, dpc; approximately 42 days before the expected farrowing date), pregnant sows allocated to the Vac/Ch group were vaccinated intramuscularly with 2.0 mL of a PRRSV-2 modified-live vaccine (MLV; PERSOPORC®, CEVA Santé Animale; Lot No. 24PRRSLG01; Expiry date: 05-FEB-2026). In parallel, animals in the UnVac/Ch and UnVac/UnCh groups received a placebo treatment consisting of 2.0 mL of phosphate-buffered saline (PBS; 0.01 M, pH 7.4) administered intramuscularly.

At 0 dpc (21 days before the expected farrowing date), pregnant sows assigned to the Vac/Ch and UnVac/Ch groups were intranasally challenged with 6 mL of tissue culture supernatant containing PRRSV-2 (strain SNUVR090851; second passage in porcine alveolar macrophages) at a dose of 10^4^ TCID₅₀/mL. In parallel, animals in the UnVac/UnCh group were administered 6 mL of virus-free cell culture supernatant via the same route as negative controls. After inoculation, sows in the Vac/Ch and UnVac/Ch groups were randomly distributed into two of the three available isolation rooms, ensuring that each room included at least one sow from each group. The UnVac/UnCh group was assigned to the third isolation room. Each isolation facility consisted of four separate pens, and all animals were maintained individually. For subsequent statistical analysis, the individual sow was considered the experimental unit.

Blood samples were collected from all pregnant sows by jugular venipuncture at four time points: −42 dpc (42 days before the expected farrowing date), 0 dpc (21 days before the expected farrowing date), 7 dpc (14 days before the expected farrowing date), and 21 dpc (parturition). For stillborn piglets, a comprehensive necropsy was conducted, and tissue samples were obtained from the lung, lymph nodes, heart, tonsil, and thymus.

### Reproductive performance

After challenge, all pregnant sows were observed daily for rectal temperature, and measurements were consistently performed by the same trained personnel. Farrowing reproductive data were recorded, including total litter size, numbers of live-born piglets, stillbirths, mummified fetuses, and piglets with low birth weight (<1 kg), both at delivery and at weaning on postnatal day 21. Farrowing was designated as premature when it occurred prior to gestational day 111.

### Quantification of PRRSV RNA in blood and tissue

Serum samples collected from pregnant sows were tested for the presence of PRRSV RNA. Two newborn piglets from each sow were randomly selected, and serum samples were obtained to determine the number of viral genomic copies. Fetal tissues of stillborn from sows in the 3 groups were also tested for the presence of PRRSV RNA. Total RNA was extracted and analyzed by RT-PCR targeting PRRSV-2, as described previously (10–12).

### Serology

Serum samples were tested for PRRSV-specific antibodies using a commercial ELISA kit (HerdChek PRRS X3 Ab Test, IDEXX Laboratories Inc., Westbrook, ME, USA). Serum virus neutralization (SVN) assays were additionally performed against the PRRSV-2 challenge strain, according to previously described methods (13). Samples were regarded as positive for neutralizing antibodies (NA) when the titer was greater than 2.0 (log₂) (14).

### Enzyme-linked immunospot assay

The frequency of PRRSV-specific interferon-*γ* secreting cells (IFN-γ-SC) in peripheral blood mononuclear cells (PBMC) was determined using an enzyme-linked immunospot (ELISPOT) assay, following established protocols (15, 16). Background activity from unstimulated negative-control wells was consistently low (≤5 spots per well) and was subtracted from the counts obtained in PRRSV-stimulated wells. Spot detection and enumeration were carried out using an automated ELISPOT reader (AID ELISPOT Reader, AID GmbH, Strassberg, Germany). All assays were conducted in duplicate to ensure reliability of the measurements.

### Statistical analysis

Prior to statistical analysis, data obtained from RT-PCR and serum virus neutralization assays were logarithmically transformed to reduce variance heterogeneity and normalize skewed distributions, using base-10 and base-2 transformations, respectively. Differences among the three treatment groups in serum PRRSV RNA levels, reproductive performance parameters, serological responses, and ELISPOT outcomes at each sampling point were analyzed using one-way analysis of variance (ANOVA). When ANOVA results indicated statistical significance, Tukey’s *post hoc* test was applied for pairwise comparisons with adjustment for multiple testing. For neutralizing antibody titers, the Shapiro–Wilk test indicated a deviation from normality; therefore, the nonparametric Kruskal–Wallis test was used. If significant group effects were observed, pairwise comparisons were subsequently performed using the Mann–Whitney U test with Tukey’s adjustment. Statistical significance was defined as *p* < 0.05.

## Results

### Reproductive performance

All pregnant sows in the vaccinated-challenged (Vac/Ch) and unvaccinated-unchallenged (UnVac/UnCh) groups carried their pregnancies to full term, farrowing between 113 and 115 days of gestation. Sows in the unvaccinated-challenged (UnVac/Ch) group farrowed prematurely, with gestation lengths ranging from 107 to 114 days. Moreover, sows in the vaccinated-challenged (Vac/Ch) and unvaccinated-unchallenged (UnVac/UnCh) groups produced significantly (*p* < 0.05) more live-born and weaned piglets, and significantly fewer stillborn piglets, compared with those in the unvaccinated-challenged group (Table 1). Throughout gestation, no systemic or local adverse reactions associated with vaccination were observed, confirming the safety of the vaccine.

**Table 1:** Reproductive parameters (mean ± standard deviation) of sows among 3 groups.

Parameters	Vac/Ch	UnVac/Ch	UnVac/UnCh
Gestation length	113.75 ± 0.5	111 ± 3.16	114.25 ± 0.5
Total born	14.5 ± 2.08	15 ± 1.41	14.5 ± 1.29
Live-born	12 ± 2.65^a^	6.75 ± 2.99^b^	13.5 ± 1.29^a^
Stillborn	2.25 ± 1.26^b^	8 ± 4.32^a^	1 ± 0.82^b^
Mummified	0 ± 0	0.5 ± 0.58	0 ± 0
Light (< 1 Kg)	0.25 ± 0.5	1.5 ± 1.91	0 ± 0
Splay-leg	0 ± 0	0.25 ± 0.5	0 ± 0
Weaned	11.25 ± 1.5^a^	5 ± 2.45^b^	12 ± 0.82^a^

Different superscripts (a, and b) indicate significant (*p* < 0.05) difference among 3 groups.

### Quantification of PRRSV RNA in sera and fetal tissues

**Figure 1:**
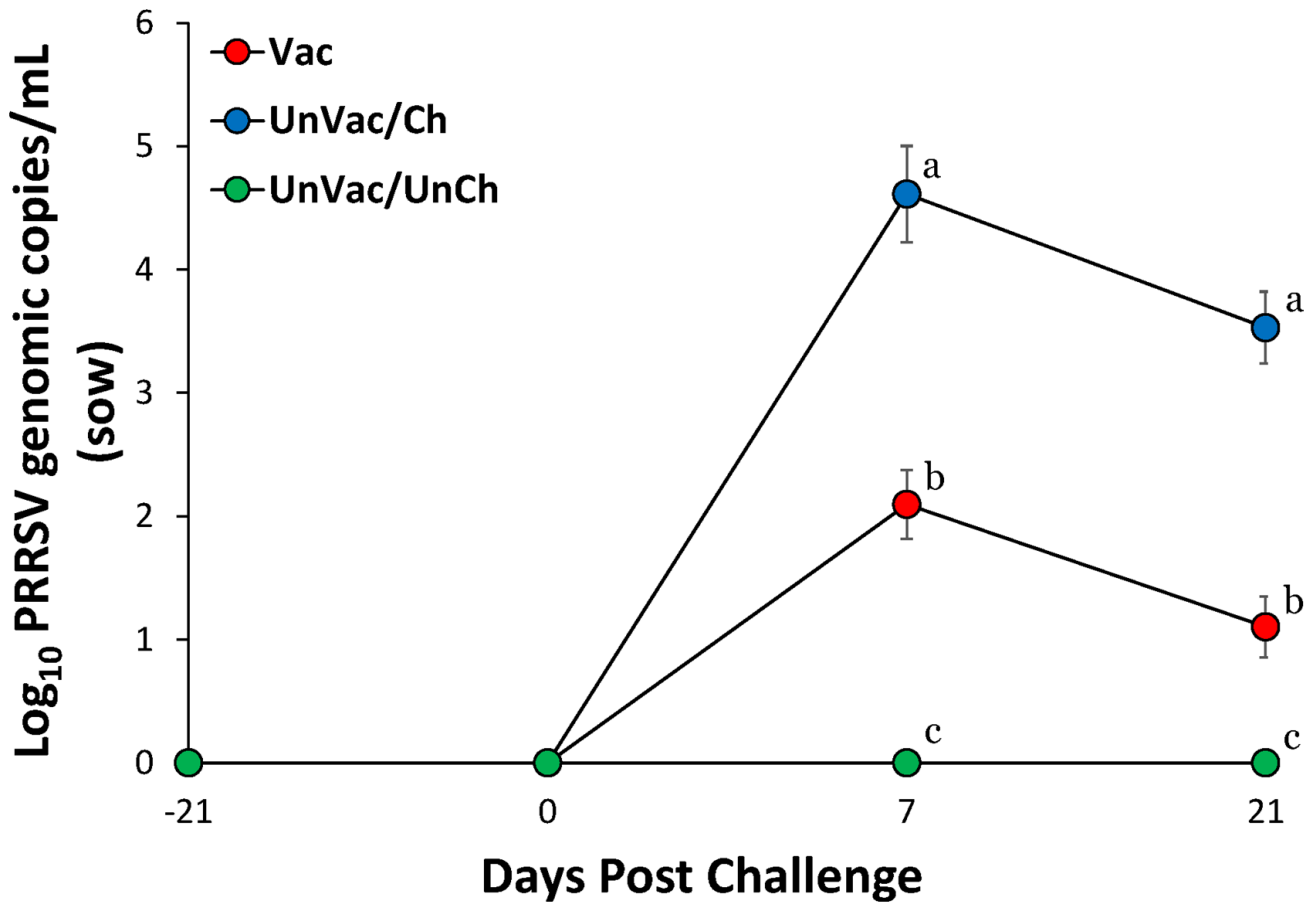
Mean values of the genomic copy number of PRRSV-2 RNA in serum in sows. Variation is expressed as the standard deviation. Different superscripts (a, b, and c) indicate significant (*p* < 0.05) difference among 3 groups.

**Figure 2:**
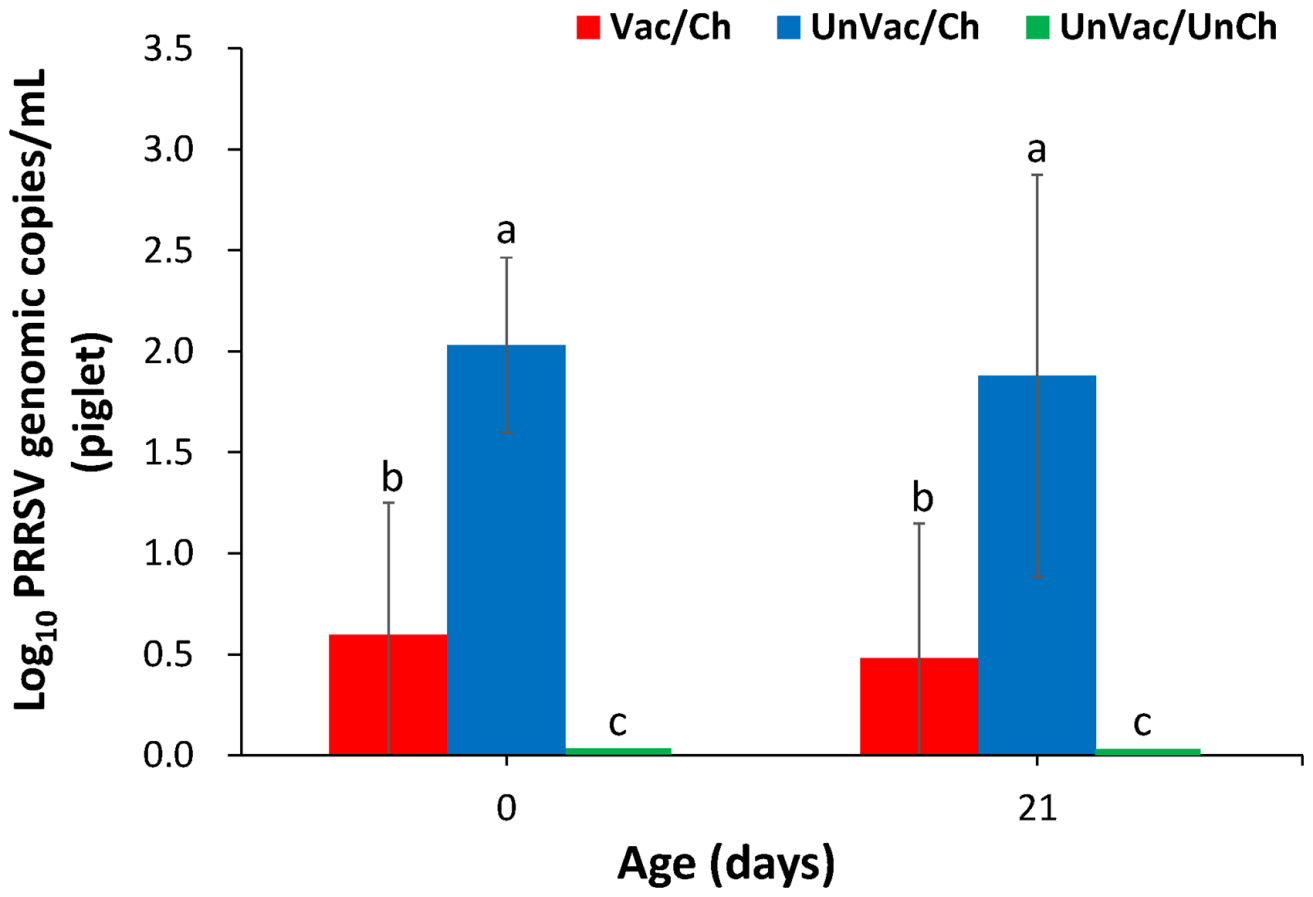
Mean values of the genomic copy number of PRRSV-2 RNA in serum in piglets. Variation is expressed as the standard deviation. Different superscripts (a, b, and c) indicate significant (*p* < 0.05) difference among 3 groups.

All pregnant sows tested negative for PRRSV at both vaccination (−42 dpc, 42 days before the expected farrowing date) and challenge (0 dpc, 21 days before the expected farrowing date), confirming their PRRSV-free status at the start of the study. At 7 and 21 dpc, sera from vaccinated-challenged sows had significantly (*p* < 0.05) fewer genomic copies of PRRSV-2 RNA compared with those from the unvaccinated-challenged sows. As expected, all animals in the unvaccinated-unchallenged group remained negative for PRRSV-2 throughout the experimental period (Figure 1). Similarly, piglets from vaccinated-challenged sows measured significantly (*p* < 0.05) lower PRRSV-2 RNA levels in their serum than those from unvaccinated-challenged sows at 1 and 21 days of age (Figure 2). All piglets born to unchallenged control sows (UnVac/UnCh) tested negative for PRRSV-2.

**Figure 3:**
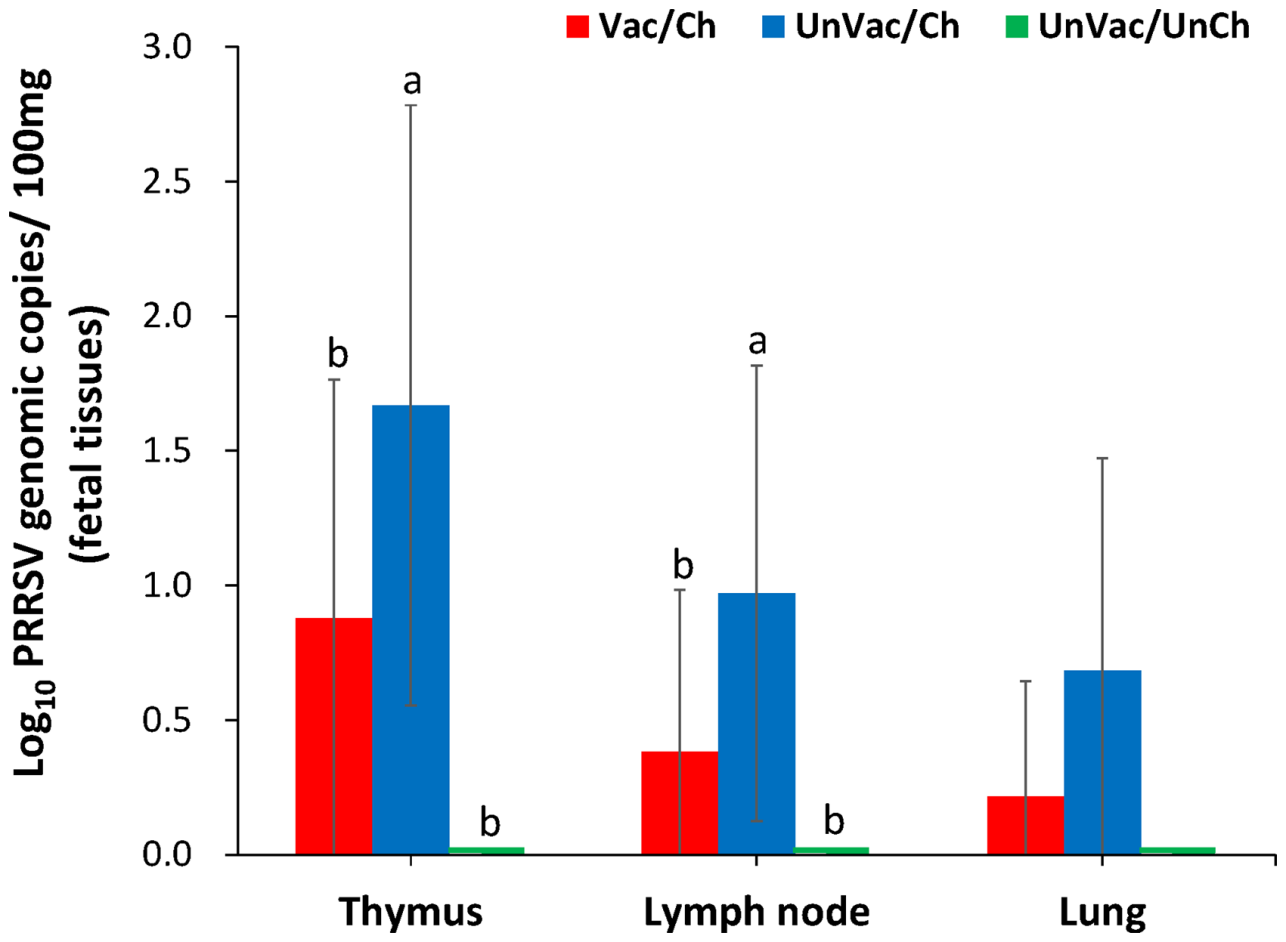
Mean values of the genomic copy number of PRRSV-2 RNA in fetal tissues of stillborn. Variation is expressed as the standard deviation. Different superscripts (a and v) indicate significant (*p* < 0.05) difference among 3 groups.

Thymus and lymph nodes of stillborn fetuses from vaccinated-challenged sows had significantly (*p* < 0.05) fewer genomic copies of PRRSV-2 RNA compared with those from the unvaccinated-challenged sows (Figure 3). All stillborn fetuses from unvaccinated control sows tested negative for PRRSV-2.

### Immune responses

**Figure 4:**
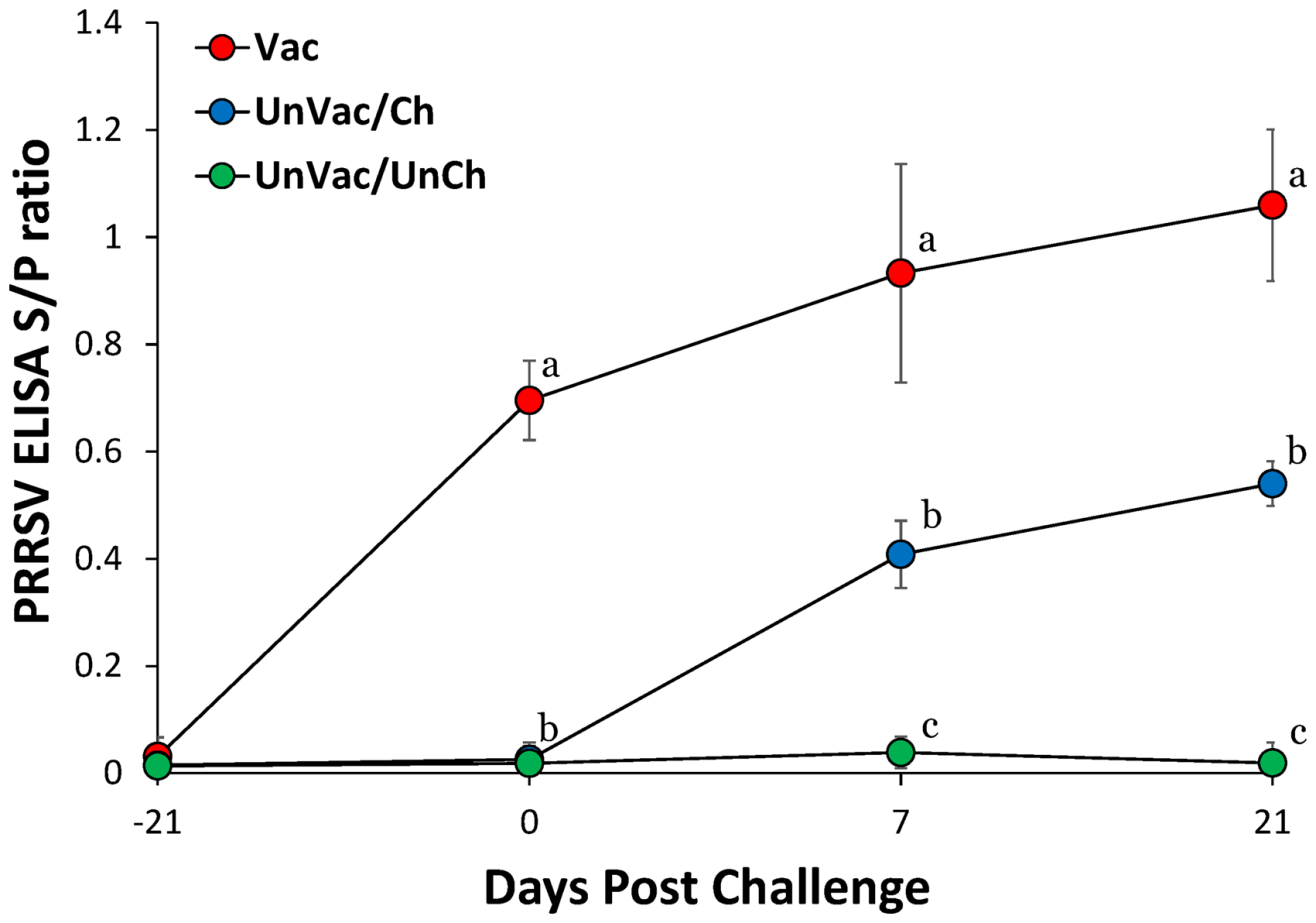
Mean values of the PRRSV ELISA S/P ratio in serum. Variation is expressed as the standard deviation. Different superscripts (a, b, and c) indicate significant (*p* < 0.05) difference among 3 groups.

At vaccination (−42 dpc, 42 days before the expected farrowing date), all pregnant sows in the three groups were seronegative, confirming their PRRSV-free status. At 0, 7, and 21 dpc, vaccinated-challenged sows developed significantly (*p* < 0.05) higher PRRSV-specific antibody titers compared with unvaccinated-challenged sows, while unvaccinated-unchallenged sows remained negative throughout the study (Figure 4). Neutralizing antibody titers against PRRSV-2 were undetectable in all three groups during the experimental period.

**Figure 5:**
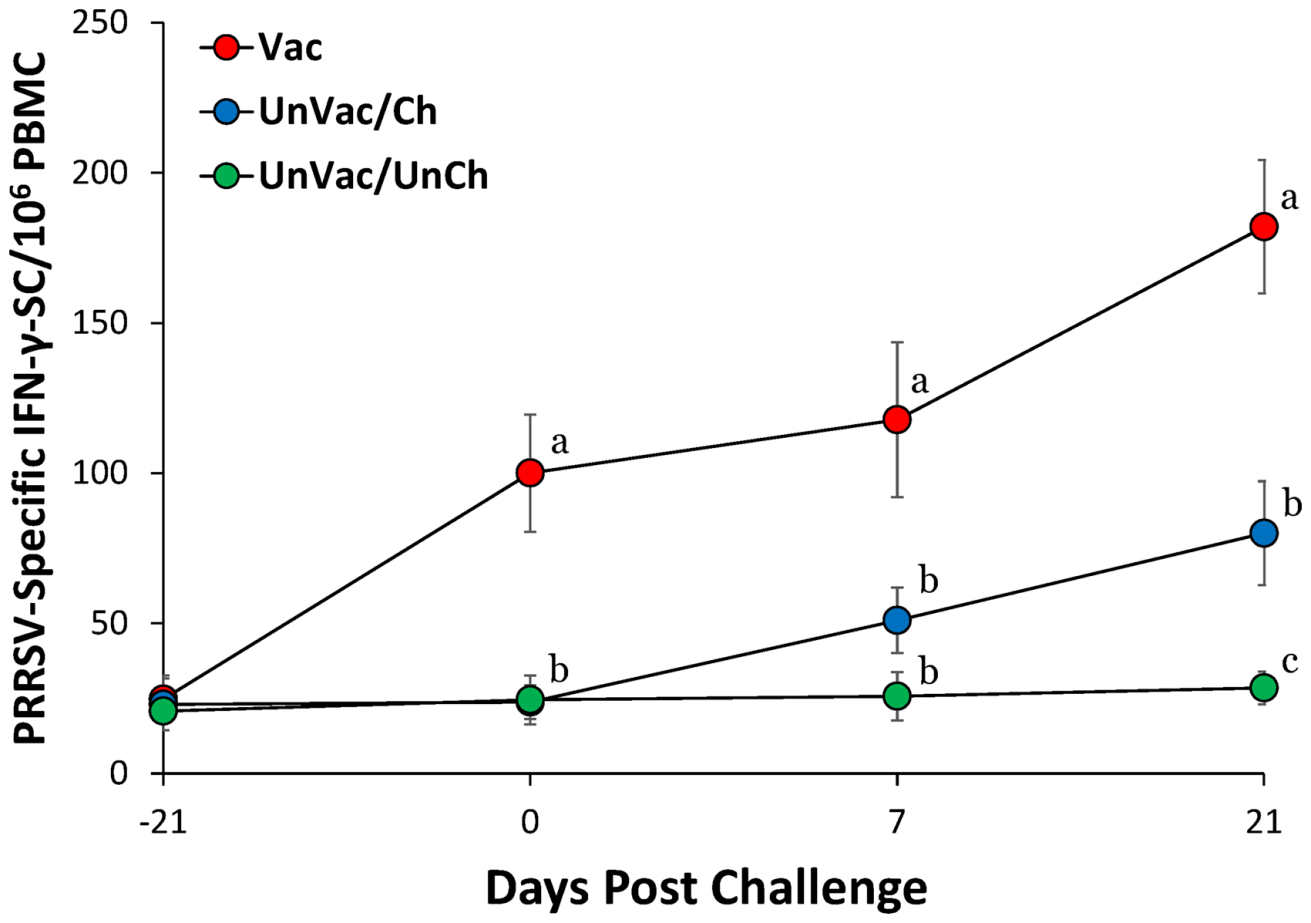
Frequency of PRRSV-2 specific IFN-*γ*-SC/10^6^ in peripheral blood mononuclear cells (PBMC). Variation is expressed as the standard deviation. Different superscripts (a, b, and c) indicate significant (*p* < 0.05) difference among 3 groups.

At vaccination (−42 dpc), none of the sows exhibited detectable PRRSV-2–specific IFN-*γ*–secreting cells (IFN-γ-SC). By 0, 7, and 21 dpc, Vaccinated-challenged sows showed a significantly (*p* < 0.05) higher frequency of PRRSV-2 specific IFN-γ-SC compared with unvaccinated-challenged sows (Figure 5). No IFN-γ-SC responses were observed in unvaccinated-unchallenged sows throughout the experiment.

## Discussion

The results of this study demonstrate that the PRRSV-2 MLV vaccine evaluated herein provides effective protection against a heterologous challenge in pregnant sows. Vaccinated animals exhibited improved reproductive performance, most notably reflected by an increased number of live-born piglets and a reduced incidence of stillbirths compared with unvaccinated controls. These improvements are particularly relevant in the context of Korea, where reproductive failure has been a major contributor to economic losses during recent PRRS outbreaks. From a practical perspective, our findings indicate that vaccination of breeding sows not only enhances reproductive outcomes but also confers substantial economic benefits to the swine industry.

The present findings clearly demonstrate that maternal vaccination against PRRSV provides dual benefits: direct protection of the dam and indirect protection of the offspring. Vaccinated sows showed significantly lower levels of viremia compared with unvaccinated controls, and this maternal advantage was transmitted to their progeny, as piglets from vaccinated sows also exhibited markedly reduced viremia. Furthermore, a significant correlation was observed between this decreased in piglet viremia which reduced the severity of PRRSV-induced pneumonia (17). These results suggest that lowering blood viral load is a critical factor in mitigating pulmonary lesions and, consequently, improving survival prospects in piglets born to vaccinated sows. Thus, a focus on viremia reduction can be considered key in determining the clinical outcomes following PRRSV infection.

To date, no commercially available vaccine has been demonstrated the full prevention of transplacental transmission or elimination of reproductive failure associated with PRRSV infection in sows (18–21). Nevertheless, vaccination of pregnant sows can reduce transplacental transmission, which is important implications for reproductive PRRS control. Reduction of vertical transmission is a critical factor of reproductive failure and neonatal mortality (22). Sow vaccination lower viral blood loads in both dams and their offspring, thus improving herd health, productivity, and economic benefits of swine production systems.

Neutralizing antibodies are typically induced late and at relatively low titers (23). Moreover, PRRSV viremia often resolves even before neutralizing antibodies become detectable in both infected and vaccinated pigs (24, 25, 26). In the present study, although neutralizing antibodies were not detected during the experimental period, vaccinated sows and their offspring exhibited reduced viremia accompanied concomitantly by an increase in frequencies of IFN-*γ*–SC. These findings indicate that viral clearance in sows may depend on vaccine-induced cellular immune responses rather than humoral immunity. Previous studies have demonstrated that virus-specific T-cell responses, particularly IFN-*γ* production, are essential for controlling PRRSV replication and mitigating disease severity (23, 24). Nevertheless, as the present study evaluated immune responses solely based on neutralizing antibody titers, ELISA S/P ratios, and IFN-γ–SC counts, further studies analyzing IFN-γ secretion kinetics and CD4^+^/CD8^+^ T-cell dynamics are warranted to elucidate the detailed protective cellular mechanisms induced by vaccination.

PRRSV antibodies measured by ELISA are not considered protective antibodies (23). Maternally derived antibodies of PRRSV, transferred from sows to piglets, typically decay around 3 weeks of age (27). Therefore, vaccination of sows against PRRSV does not prevent respiratory disease caused by PRRSV in their offspring. Consequently, to ensure protection of piglets against PRRSV-associated respiratory disease, vaccination at 3 weeks of age is recommended.

In the present study, vaccinated animals remained positive for vaccine virus for up to 2 weeks post-vaccination. This limited duration of viremia substantially reduces the likelihood of horizontal transmission to unvaccinated animals, thereby confirming the safety of the PRRSV-2 MLV tested in this study. Furthermore, sequencing of the open reading frame 5 gene from PRRSV detected in pigs is able to differentiate vaccine-derived strains from circulating wild type field viruses, providing an additional tool for monitoring vaccine safety under field conditions.

The limitation of the present study is the relatively small sample size, which may have reduced the statistical power of the analysis. However, the number of animals used in the present study was restricted in accordance with the recommendations of the Seoul National University Institutional Animal Care and Use Committee to minimize unnecessary use of pregnant sows. In future field trials, the number of vaccinated sows should be increased to address this limitation and to further validate the efficacy observed in this controlled experimental challenge study.

The present experimental challenge study of maternal vaccination carries important clinical implications. In many Asian countries, including Korea, substantial economic losses in breeding herds are attributable to reproductive failure caused by PRRSV-2 strains of lineage 1. Given that the vaccine evaluated in this study conferred protective efficacy against lineage 1 PRRSV-2, it may play a pivotal role in reducing reproductive losses and alleviating the economic losses of the disease in commercial swine production systems across the Asian countries. Although the currently available and tested PRRSV-2 MLV vaccines did not induce sterilizing immunity and cannot completely prevent PRRSV infection, as evaluated against heterologous challenge, they remain the most effective control measure to-date. They effectively reduced viremia and improved reproductive outcomes, highlighting their essential role in herd-level PRRSV control.

## Data Availability

The original contributions presented in the study are included in the article/supplementary material, further inquiries can be directed to the corresponding author.
